# Multiscale computational model of Achilles tendon wound healing: Untangling the effects of repair and loading

**DOI:** 10.1371/journal.pcbi.1006652

**Published:** 2018-12-14

**Authors:** Kellen Chen, Xiao Hu, Silvia S. Blemker, Jeffrey W. Holmes

**Affiliations:** 1 Department of Biomedical Engineering, University of Virginia, Charlottesville, VA, United States of America; 2 Department of Mechanical and Aerospace Engineering, University of Virginia, Charlottesville, VA, United States of America; 3 Department of Orthopaedic Surgery, University of Virginia, Charlottesville, VA, United States of America; 4 Department of Medicine, University of Virginia, Charlottesville, VA, United States of America; Stanford University, UNITED STATES

## Abstract

Mechanical stimulation of the healing tendon is thought to regulate scar anisotropy and strength and is relatively easy to modulate through physical therapy. However, *in vivo* studies of various loading protocols in animal models have produced mixed results. To integrate and better understand the available data, we developed a multiscale model of rat Achilles tendon healing that incorporates the effect of changes in the mechanical environment on fibroblast behavior, collagen deposition, and scar formation. We modified an OpenSim model of the rat right hindlimb to estimate physiologic strains in the lateral/medial gastrocnemius and soleus musculo-tendon units during loading and unloading conditions. We used the tendon strains as inputs to a thermodynamic model of stress fiber dynamics that predicts fibroblast alignment, and to determine local collagen synthesis rates according to a response curve derived from *in vitro* studies. We then used an agent-based model (ABM) of scar formation to integrate these cell-level responses and predict tissue-level collagen alignment and content. We compared our model predictions to experimental data from ten different studies. We found that a single set of cellular response curves can explain features of observed tendon healing across a wide array of reported experiments in rats–including the paradoxical finding that repairing transected tendon reverses the effect of loading on alignment–without fitting model parameters to any data from those experiments. The key to these successful predictions was simulating the specific loading and surgical protocols to predict tissue-level strains, which then guided cellular behaviors according to response curves based on *in vitro* experiments. Our model results provide a potential explanation for the highly variable responses to mechanical loading reported in the tendon healing literature and may be useful in guiding the design of future experiments and interventions.

## Author summary

Tendons and ligaments transmit force between muscles and bones throughout the body and are comprised of highly aligned collagen fibers that help bear high loads. The Achilles tendon is exposed to exceptionally high loads and is prone to rupture. When damaged Achilles tendons heal, they typically have reduced strength and stiffness, and while most believe that appropriate physical therapy can help improve these mechanical properties, both clinical and animal studies of mechanical loading following injury have produced highly variable and somewhat disappointing results. To help better understand the effects of mechanical loading on tendon healing and potentially guide future therapies, we developed a computational model of rat Achilles tendon healing and showed that we could predict the main effects of different mechanical loading and surgical repair conditions reported across a wide range of published studies. Our model offers potential explanations for some surprising findings of prior studies and for the high variability observed in those studies and may prove useful in designing future therapies or experiments to test new therapies.

## Introduction

Many mechanically loaded tissues including skin, tendon, ligament, and heart respond to injury by forming a collagen-rich scar. Normally, tendons are comprised of highly aligned collagen fibers that help transmit forces between muscles and bones throughout the body and bear high loads. The Achilles tendon in particular can be exposed to loads up to 70 MPa, compared to 30 MPa in most other tendons [1]. These high loads often lead to injury, with Achilles tendon ruptures accounting for up to 45% of all tendon ruptures [2] and afflicting up to 2.5 million annually [3,4]. Many who suffer from an Achilles tendon rupture never regain complete function, especially because healing tendons form scar with reduced collagen fiber organization and stiffness compared to uninjured tendons [5].

While there seems to be general agreement that mechanical stimulation of the healing tendon, such as during physical therapy, influences scar mechanical properties [6], current treatments for patients with an Achilles tendon rupture have produced variable results [1,3,7–9]. To better understand the impact of loading, rat animal models of Achilles tendon rupture have been utilized so that scar tissue can be excised to determine quantitative biomechanical scar properties. Unfortunately, these studies have also led to a wide variety of results, with mechanical loading sometimes appearing to increase, but at other times appearing to decrease, tendon properties such as stiffness or rupture strength. One of the potential explanations for this variability could be due to differences in mechanics during healing, which could be altered through unloading (e.g. cast immobilization) or loading (e.g. free cage activity) of the tendon [10–14] as well as through the choice to repair the tendon with suture versus allowing natural healing without repair [12,15]. In this work, we developed a multiscale computational model of the healing rat Achilles tendon that integrates information about how local mechanics influences cellular alignment and collagen remodeling to predict the effects of various repair and loading protocols on tendon structure. We found that the multiscale model predicted the major observed trends in the evolution of tissue-level scar properties across a wide variety of published rat studies. Furthermore, the model simulations identified a potential mechanism underlying the apparently paradoxical finding that mechanical loading enhances collagen alignment in unrepaired Achilles tendons yet decreases it in repaired tendons.

## Results and discussion

**Fig 1:**
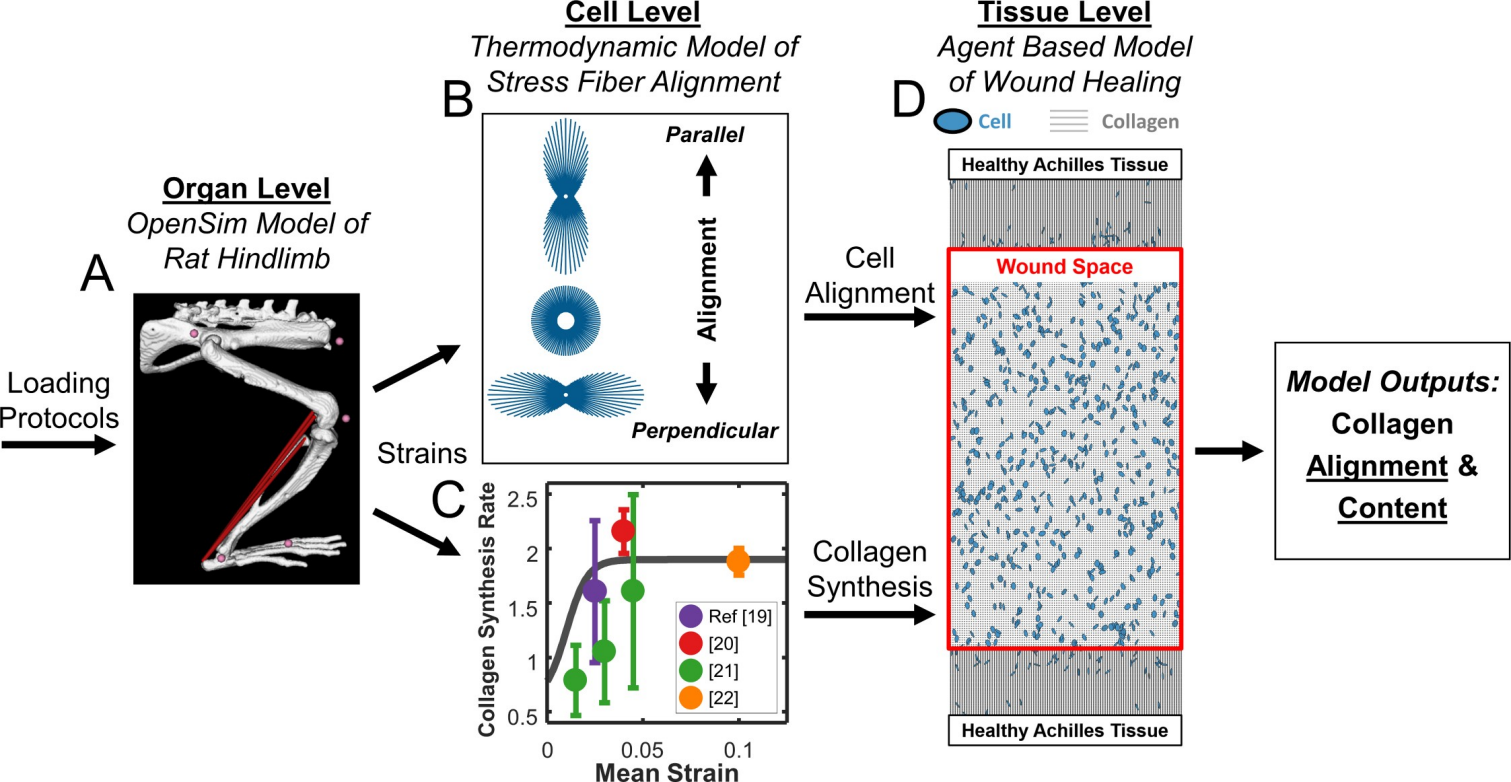
Flowchart depicting multiscale model. **(A)** OpenSim model of rat hindlimb adapted from Johnson 2008 [16] simulates strains in the injured Achilles tendon. **(B)** Cell model predicts alignment based on the mechanical environment. **(C)** Fitted mean strain versus collagen I production curve (**grey line**) from four independent studies governs collagen production. **(D)** Agent-based model (ABM) of wound healing integrates migration, alignment, and collagen deposition by cells (**blue**) to predict scar collagen content and alignment (**grey**).

Our multiscale computational model simulated mechanics and associated responses at multiple scales. At the organ level, we simulated various healing and loading conditions in a musculoskeletal model of the right rat hindlimb [16] implemented in OpenSim [17] to estimate associated strains in the tendon (Fig 1A). At the cellular level, these strains affected cellular behavior in two ways (Fig 1B and 1C). First, cyclic strains determined cell alignment according to a thermodynamic model of stress fiber dynamics developed and validated against *in vitro* experiments by our group [18] (Fig 1B). Second, mean strains modulated fibroblast collagen synthesis according to a relationship fitted to data from multiple published studies (Fig 1C) [19–22]. At the tissue level, we used an agent-based model (ABM) of wound healing similar to one published previously by our group [23] to integrate these cellular behaviors and predict the evolving collagen structure (Fig 1D).

### Effect of unloading and loading on unrepaired Achilles tendons

**Fig 2:**
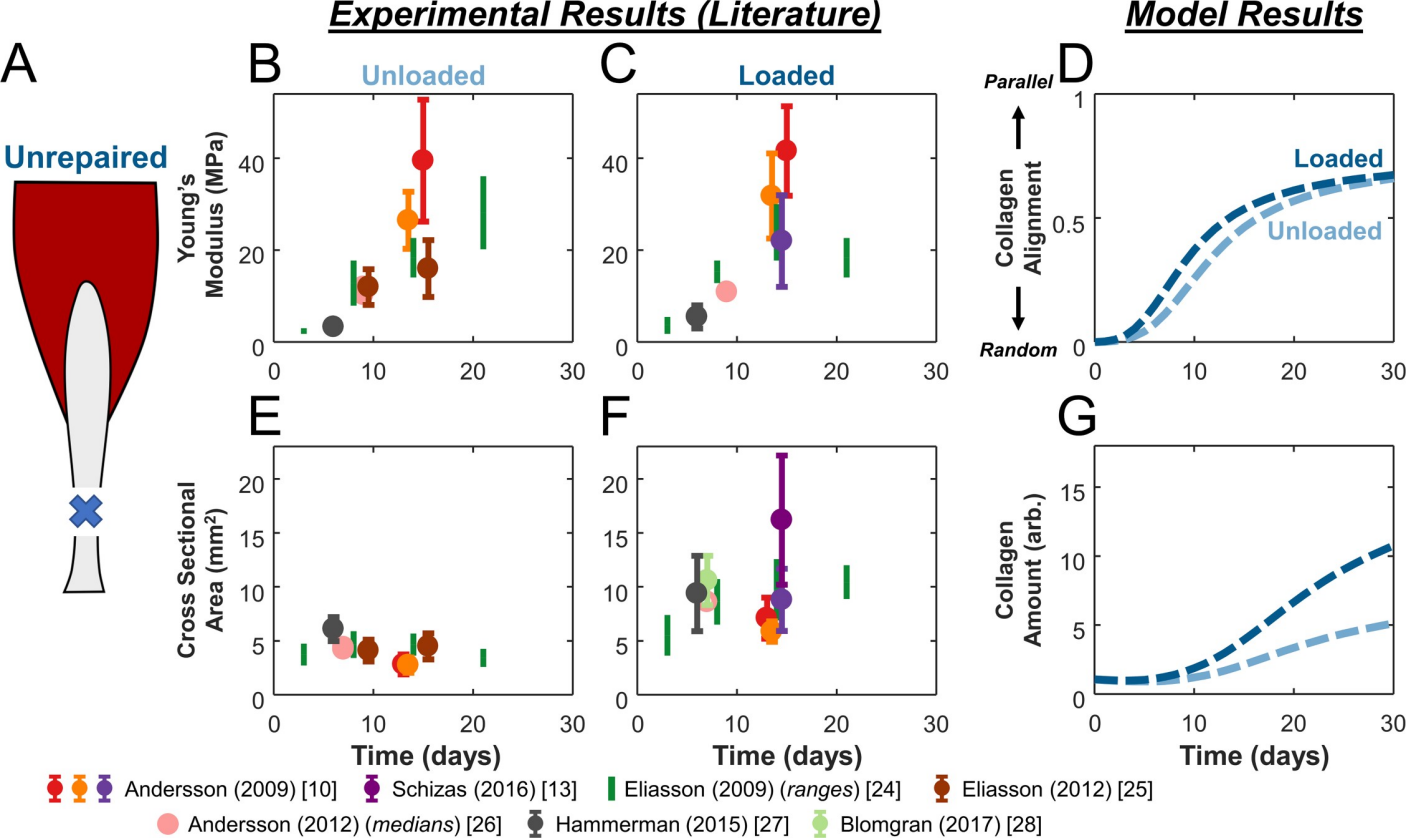
Model predictions of collagen alignment and content match trends in tissue-level properties reported in the literature during healing of unrepaired tendons. **(A)** Schematic of rat Achilles tendon transection injuries left to heal naturally. **(B,C,E,F)** We plotted data from studies **(various colors)** in which tendons healed unloaded **(B,E)** or loaded **(C,F)**. We compared our predictions of collagen alignment **(D)** and content **(G)** to experimentally measured Young’s Moduli **(B,C)** and cross-sectional areas **(E,F)**, see text for details.

We searched the literature for studies of transected Achilles tendons in rats, the most common animal model used to mimic an Achilles tendon rupture. These studies used a variety of time courses and mechanical loading protocols to treat the rats during the wound healing process. First, we focused on studies that tested the effects of natural healing of unrepaired, transected tendons and imposed unloading by either botulinum toxin (Botox) injection into the gastrocnemius muscle or tail suspension [10,24–26] or loading by allowing the rat to freely walk around its cage [10,13,27,28] (Fig 2A). Because no studies quantified both collagen alignment and total collagen mass, we then narrowed our search to include only studies that reported at least one of two quantitative measures that could act as surrogates for model comparisons. Based on the strong reported correlation between measured collagen alignment and intrinsic material properties of the tissue [6,14], we compared measured values of Young’s modulus (E_y_ in MPa) to the levels of collagen alignment predicted by our model. Similarly, based on previous studies showing that collagen concentration and tendon CSA rise in parallel [24,29,30], as well as the fact that tissue mass increases with tissue volume, we compared measured values of tendon cross-sectional area (CSA in mm^2^) to the total collagen content predicted by the model. Despite considerable variability among the reported values of these metrics, two clear trends were apparent in the data. First, E_y_ started near 0 MPa at 3 days and rose to about 20–40 MPa at 14 days in both unloaded and loaded conditions (Fig 2B and 2C). Second, CSA values in unloaded conditions remained at around 5 mm^2^ (Fig 2E), while loading increased CSA over time to around 10–20 mm^2^ at day 14 (Fig 2F).

**Fig 3:**
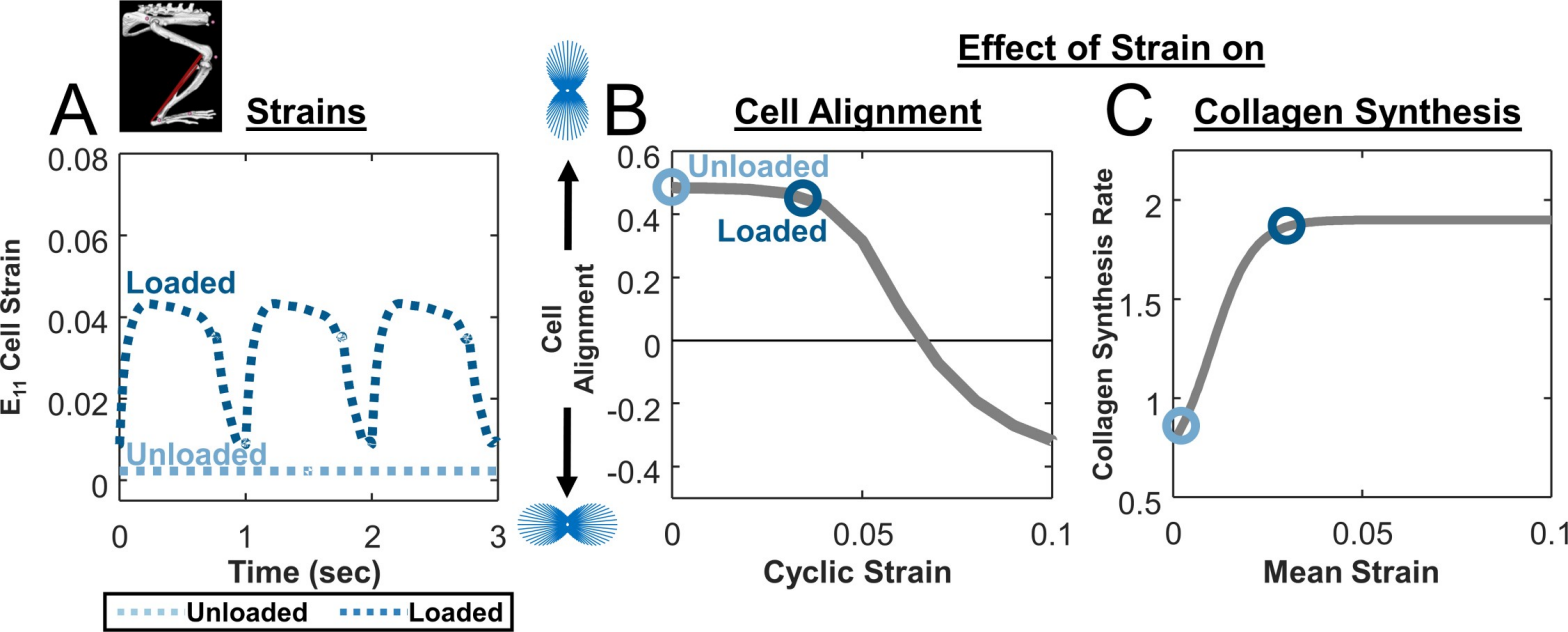
Effect of strain on cellular alignment and collagen synthesis. **(A)** Estimated cellular strain profiles derived from tendon strain profiles predicted by modified rat hindlimb model. **(B)** Cellular alignment response curve generated by model of stress fiber dynamics in response to cyclic stretching at various magnitudes and a frequency of 1 Hz with a 1h on, 1h off repeating cycle during waking hours (**gray line**); open circles show the operating points on this curve for simulated unloading (**light blue circle**) and loading conditions (**dark blue circle**) in unrepaired tendons. **(C)** Response curve showing the effect of mean strain on fibroblast collagen synthesis rate (**gray line**), again overlaid with circles showing the operating points for the specific cases simulated in Fig 2.

Next, we used the model of the rat hindlimb implemented in OpenSim [16] to simulate these unloading and loading conditions and estimate strains in the healing region. We simulated unloading by fixing all joint ankles in plantar flexion, with minimum muscle activation and a 7mm gap distance between the tendon stumps [10,25,31], resulting in a predicted constant cell strain of E_11_ = 0.002 in the primary loading direction (Fig 3A). We simulated loading by prescribing joint angles and muscle activation corresponding to the rat gait cycle. Predicted strains oscillated between .009 and .043, reflecting the swing/stance phases of the gait cycle and yielding a cyclic strain amplitude of 0.034 and a mean strain of 0.030 (Fig 3A). Using our published model of stress fiber dynamics [18], both strain states produced cell alignment along the loading axis, with slightly lower predicted alignment for the loaded case (Fig 3B). Using the collagen synthesis curve we fitted to published data [32–35], mean strain from the loaded case was associated with about 2x greater collagen production than the mean strain from the unloaded case (Fig 3C).

Integrating these two cellular behaviors in the ABM component, the multiscale model predicted that the specific loading protocols we simulated should produce little difference in collagen alignment (Fig 2D) but a substantial difference in collagen content (Fig 2G). Trends in predicted alignment agreed with literature reports showing similar E_y_ for both groups at all time points (Fig 2B and 2C). Furthermore, differences in model-predicted collagen accumulation in the two loading states qualitatively matched reported differences of the tendon CSA in loaded (Fig 2F) compared to unloaded (Fig 2E) conditions.

### Effect of unloading and loading on surgically repaired Achilles tendons

**Fig 4:**
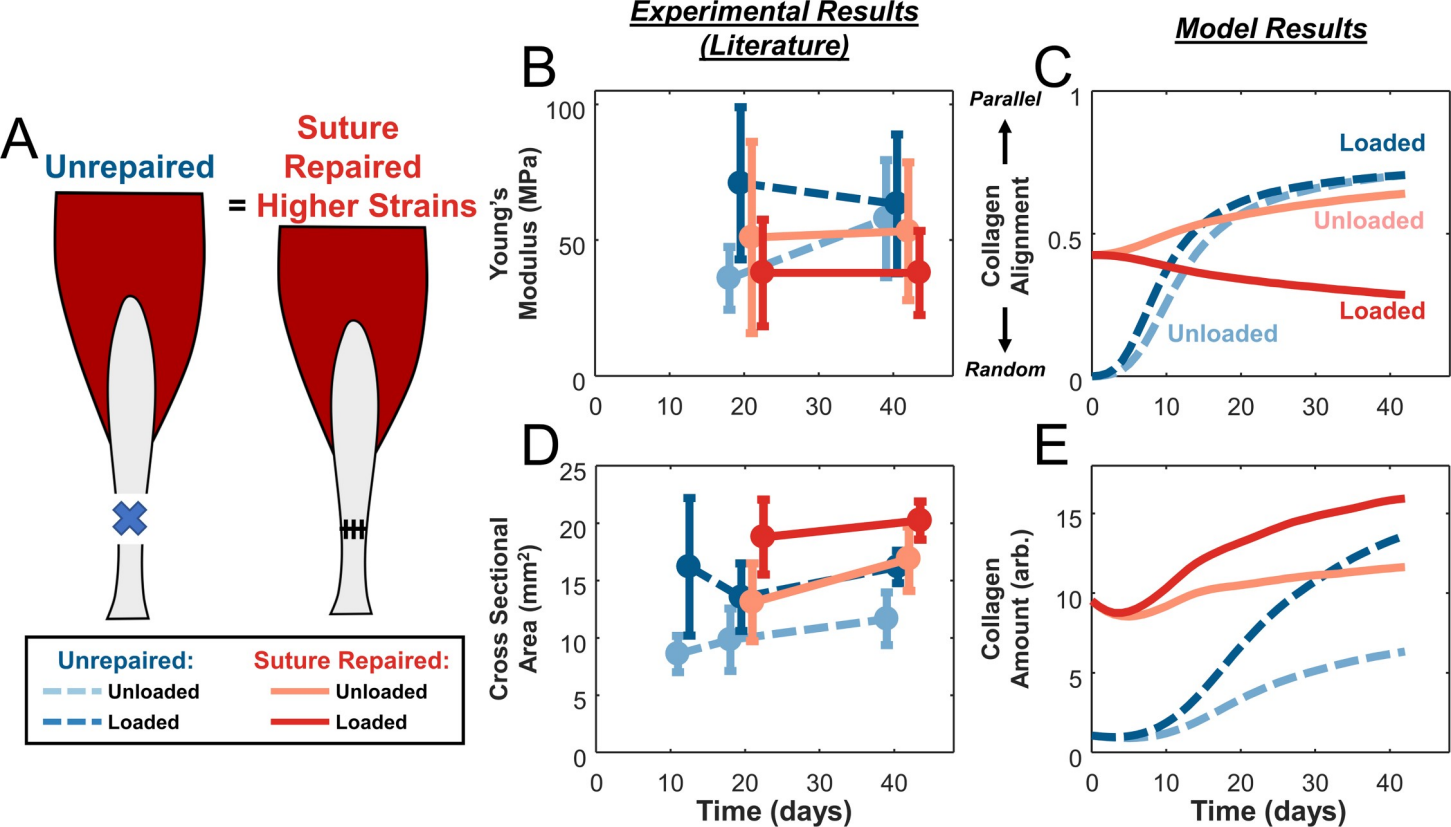
Model predictions of the effects of loading in unrepaired versus suture-repaired tendons also match experimentally observed tissue-level measures. **(A)** Schematic of rat Achilles tendon transection injuries left unrepaired or repaired with sutures. **(B,D)** Studies [12,13,36] that tested both unloading (**light color**) and loading (**dark color**) conditions in either unrepaired (**dashed blue lines**) or repaired (**solid red lines**) tendons showed a lower Young’s modulus in the loaded, suture-repaired case and similar values in the other groups **(B)**, the same trend predicted for collagen alignment by the model **(C)**. Experimental data showed higher tendon cross-sectional area with loading regardless of repair status **(D)**, matching model predictions of total collagen **(E)**.

In our next set of simulations, we explored how surgically repairing rat Achilles tendons would alter our predictions and the response to loading during healing (Fig 4A). We selected a set of studies that subjected experimental groups to either unloading with cast immobilization or loading with an exercise protocol, defined as 60 min/day treadmill exercise + free cage activity for the rest of the time, compared surgically repaired and unrepaired groups, and reported Young’s modulus or cross-sectional area (Fig 4B and 4D) [12,13,36]. For the unrepaired groups in this second set of studies, trends were similar to those shown in Fig 2, with loading enhancing CSA but not E_y_. Similar to the unrepaired groups, loading of suture-repaired tendons increased tendon CSA compared to unloading (Fig 4D). However, loading in the suture-repaired groups surprisingly reduced Young’s modulus below the values observed in any other group by the 42-day final time point (Fig 4B).

**Fig 5:**
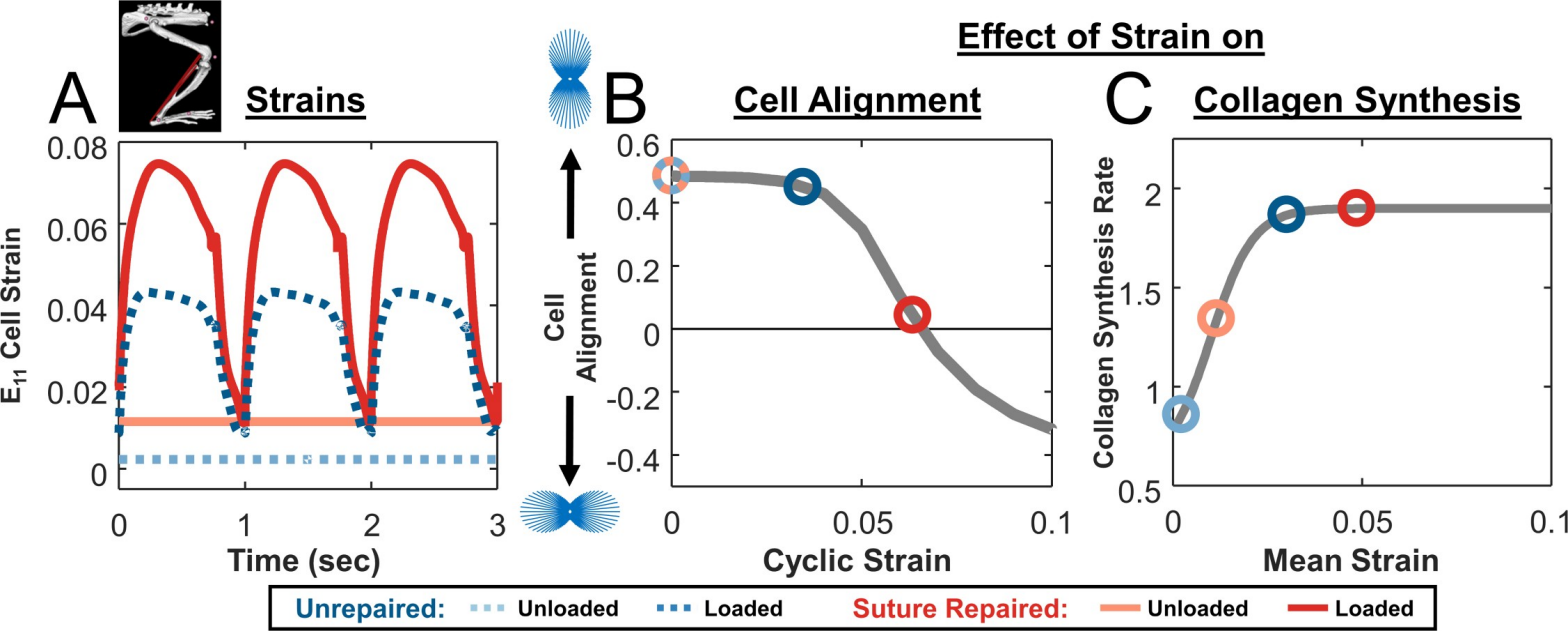
Cellular strain profiles and corresponding responses for simulations shown in Fig 4. **(A)** Estimated cellular strain profiles derived from tendon strain profiles predicted by hindlimb model in OpenSim. **(B)** Cellular alignment response curve generated by model of stress fiber dynamics (**gray line,** same as Fig 3) and operating points on that curve for unloading (**light color**) and loading (**dark color**) conditions in both unrepaired (**blue**) and suture-repaired (**red**) tendons. **(C)** Response curve showing the effect of mean strain on fibroblast collagen synthesis rate (**gray line,** same as Fig 3) and circles indicating the operating points for the different cases simulated.

**Fig 6:**
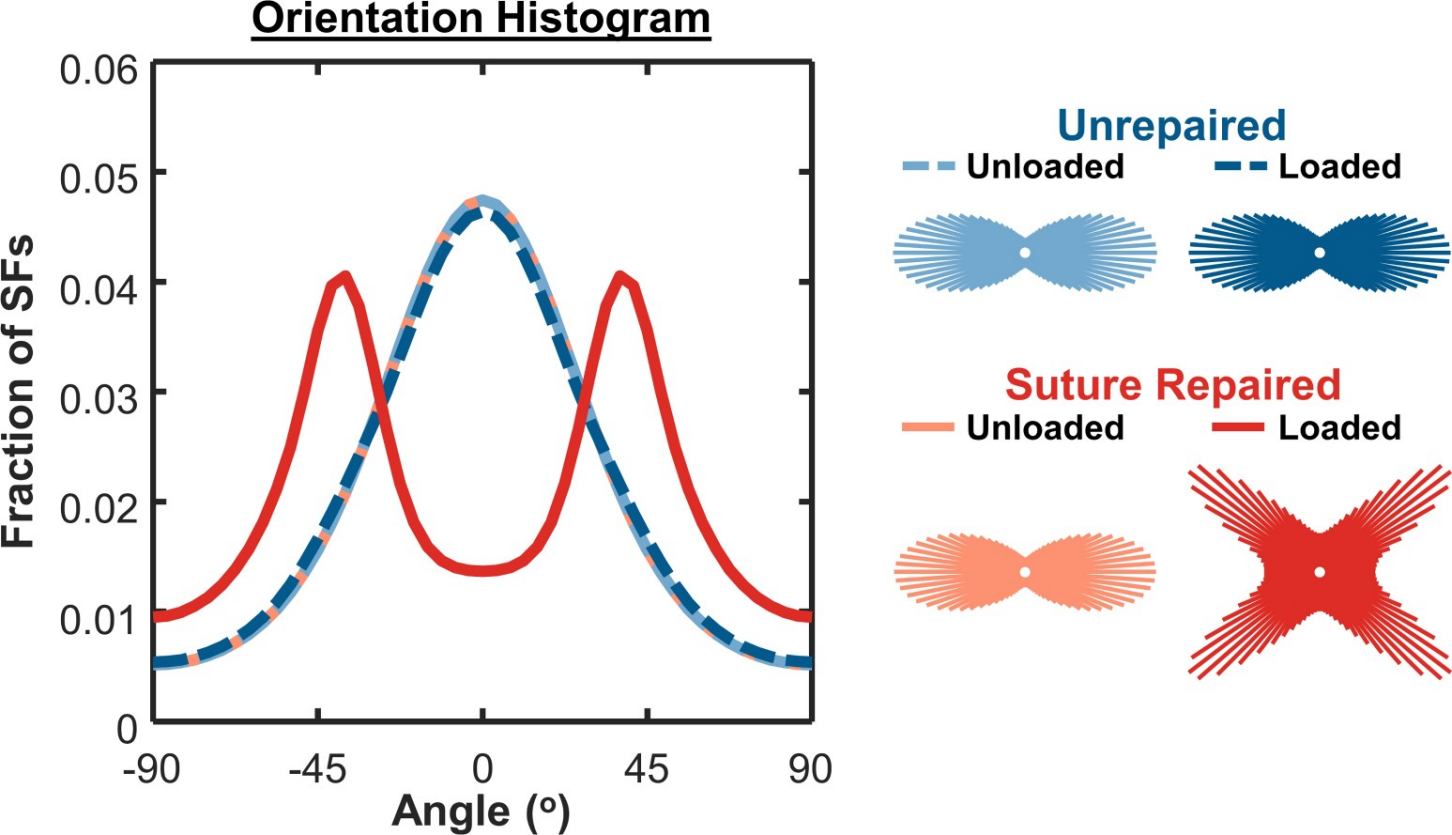
Angular histograms of cell stress fiber orientation from thermodynamic alignment model. Loaded, suture-repaired tendons exhibited lower cell alignment compared to other conditions. Insets underneath legend labels show corresponding circular histograms of stress fiber (SF) orientation.

Assuming that surgical repair eliminated the gap distance between the tendon stumps, our hindlimb simulations in OpenSim predicted higher strains for repaired conditions compared to unrepaired, with repaired-unloaded tendons experiencing a static strain of E_11_ = 0.012 and repaired-loaded tendons oscillating between 0.011 and 0.075 (cyclic strain of 0.064, mean strain of 0.049; Fig 5A). The much larger cyclic strains in the repaired-loaded group induced stress fiber disassembly along the loading axis in the cell alignment model, resulting in cells that were nearly randomly oriented (Fig 5B); the differences in predicted stress fiber orientation distributions for these four cases are shown in Fig 6. On the other hand, the large mean strains in the repaired-loaded group led to a higher rate of collagen synthesis in this group compared to the other conditions simulated (Fig 5C). Integrating these predictions using the ABM component, overall our multiscale model correctly matched the apparently paradoxical reports that repaired, loaded tendons have the lowest collagen alignment of any of these four conditions (Fig 4B and 4C) despite having the highest collagen content (Fig 4D and 4E). In other words, a single set of cellular response curves can explain features of observed tendon healing across a wide array of reported experiments in rats without fitting model parameters to any data from those experiments. Rather, the key to the predictions in our multiscale model is simulating the specific loading and surgical protocols to predict tissue-level strains, which then guide cellular behaviors according to response curves based on *in vitro* experiments.

### Effect of intermittent loading on cell alignment

**Fig 7:**
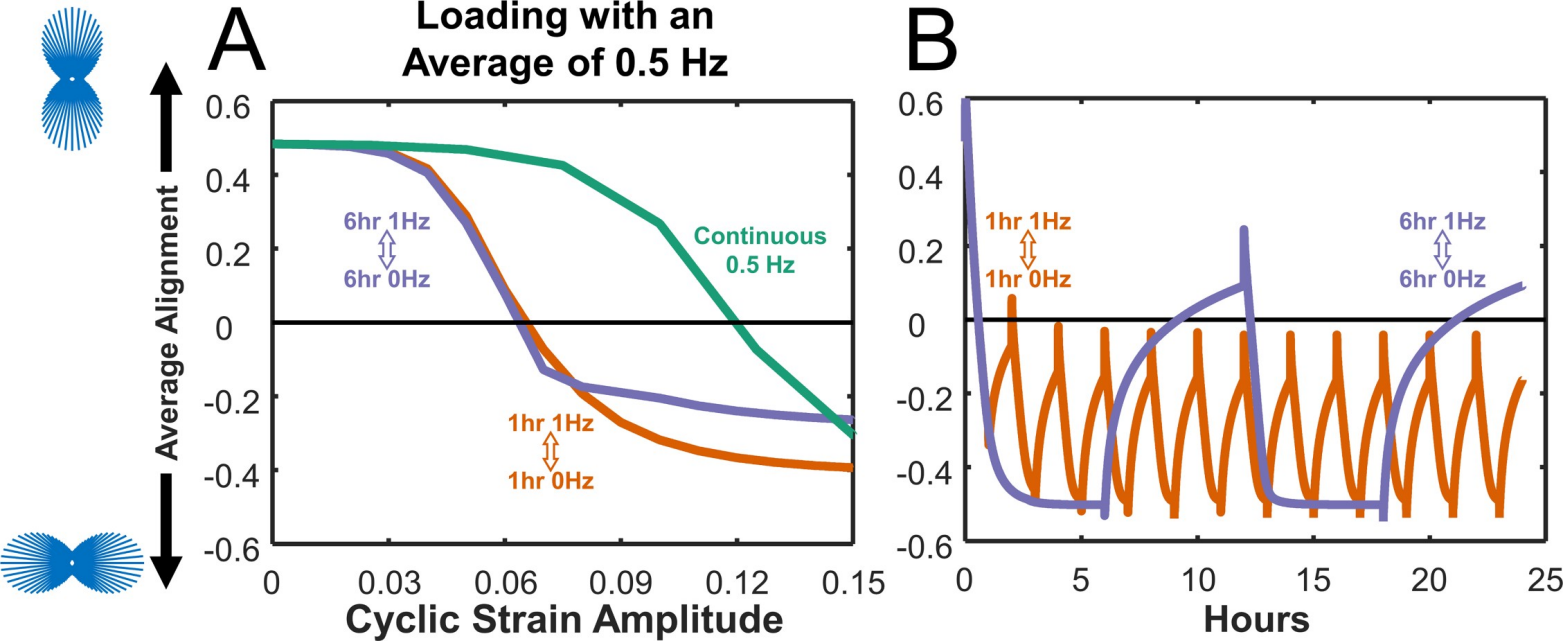
Identical average stretch frequencies result in varying cell alignment predictions using cell model. **(A)** Steady-state predicted alignment response curves for cells stretched in cycles of 6 hours at 1Hz followed by 6 hours of rest (0Hz) (**purple**), cycles of 1 hour at 1Hz followed by 1 hour of rest (**orange**), and continuous stretching at 0.5Hz (**green**). **(B)** Detailed time course of predicted alignment during 24 hours of intermittent stretch using two different duty cycles and a strain amplitude of 0.10. Each strain protocol was repeated until the difference in alignment at the end of two consecutive repetitions was less than 0.01.

Many of the studies we simulated here loaded healing tendons through unrestricted cage activity or through daily exercise added to normal cage activity. In these protocols, tendons are cyclically loaded in short bursts as the animals move about their cages, interspersed with short rest periods when they are standing still and longer rest periods when they sleep. We chose to model this situation by imposing cyclic loading at 1 Hz with a 1-hour on, 1-hour off protocol for 12 hours, followed by 12 hours of rest, and then repeating. To understand the effect of this choice on our results, we used the cell alignment model to simulate the effect of stretching with different protocols that all produced the same time-averaged frequency of 0.5Hz (Fig 7A). We compared stretching for repetitions of 6 hours at 1Hz followed by 6 hours of rest (0Hz), repetitions of 1 hour at 1Hz followed by 1 hour at rest, and continuous stretching at 0.5Hz. We simulated each repetition until steady state was reached, defined as the point when the difference in alignment at the end of two consecutive repetitions was less than 0.01 (Fig 7B). We found that the different stretching protocols produced different relationships between strain amplitude and predicted steady-state cell alignment, defined as the average order parameter over the last simulated cycle. At low strain amplitudes, all the protocols produced similar results, while at peak strains between 0.06 and 0.10 the three protocols resulted in predictions ranging from fairly strong alignment parallel to stretch (continuous stretching) to a modest degree of perpendicular alignment (1h on / 1h off and 6h on / 6h off). These differences arise from two features of the stress fiber model. First, stress fiber disassembly is triggered by high negative strain rates in the model, so much higher strains are required to influence alignment when loading is imposed at a lower frequency (Fig 7A). Second, because disassembly of stress fibers occurs on a much faster time scale than assembly, shorter durations of cyclic stretching can drive alignment down quickly, while much longer rest periods are needed to recover from each loading cycle (Fig 7B).

Our simulations of intermittent loading protocols raise the possibility that relatively minor differences in loading could have significant implications for fibroblast and collagen alignment. This could be an intriguing explanation for the surprising degree of variability we found in the literature among studies that employed apparently identical experimental protocols and outcome metrics (see Fig 2B, 2C and 2F), or even within groups in individual studies (see error bars in Fig 4B and 4D). To date, most published experiments have not tracked movement of rats with the detail required to simulate more realistic or even animal-specific loading protocols. Sams-Dodd observed that healthy rats traveled around 5700 cm within a 10 minute observation period in one of the only studies we could find that attempted to track travel distances [37]. Our simulations suggest that employing continuous movement tracking in future studies might provide additional insight into whether individual variability in activity and loading can explain some of the observed variability in tendon healing.

### Limitations

The most serious limitation of the modeling studies reported here is that we were only able to validate them through qualitative comparisons of model-predicted trends to experimentally measured surrogates. The most novel prediction of our model was the degree of collagen fiber alignment. Experimentally, collagen alignment can be directly measured from ultrasound [4,12] or polarized microscopy [38]. However, these measurements were reported in so few studies that we were forced to use a more commonly reported surrogate, the Young’s modulus (E_y_), which has been shown to correlate with the degree of alignment. It is conceivable that other factors such as collagen density that might differ between the cases simulated here might have influenced E_y_. Future studies may be able to draw on relationships between alignment and modulus such as those reported by Lake et al. [39] and Li et al. [40] to quantitatively estimate E_y_. The comparison of total collagen content to tendon cross-sectional area (CSA) could also have limitations, since experimentally the rate of collagen production influences both collagen density (as assessed by biochemical assays or picrosirius red staining) and total CSA, but few studies provide data on both simultaneously. In addition, our collagen content predictions are in arbitrary units, since the studies we used to determine the effect of stretch on collagen synthesis reported relative changes rather than absolute synthesis rates.

The simplifications we made in the rat hindlimb model could also introduce some errors in the tendon-level strains we calculated. First, we included only the musculo-tendon units that directly comprise the Achilles tendon. Second, due to a lack of information in the literature about hip and knee motion during walking in the setting of rat Achilles tendon injury, we combined healthy hip and knee with injured ankle motion [41,42]. However, we expect errors introduced by this choice to be small, since the gastrocnemius and soleus muscles do not cross the hip, the soleus does not cross the knee, and the moment arms around the ankle are larger than at the knee for the gastrocnemius. Furthermore, as long as simulated strains from repaired tendons are higher than unrepaired and strains from loading are higher than from unloading, the overall trends predicted from our model should be robust to small changes in the exact magnitude of the predicted tendon strains.

Our agent-based model assumes that cells deposit collagen aligned with their own axis, and this assumption was critical to translating cell alignment predictions from the cytoskeletal model into tissue-level predictions of collagen structure. While the exact mechanisms by which fibroblasts deposit and orient collagen *in vivo* are still being debated, the general idea that collagen ends up locally aligned with the fibroblasts that deposit it remains strongly supported in the literature [43–45]. Furthermore, we have previously shown that agent-based models incorporating this same assumption correctly predict a range of scar structures observed following myocardial infarction in various animal models under different mechanical conditions [23]. We also considered and simulated several other alternative methods of determining collagen alignment in the model but found that none could predict all the observed trends apparent in the data reviewed here. For instance, several experiments have theorized that surrounding collagen fibers could “structurally constrain” the formation of new fibers *in vivo* [46–48]. Others have demonstrated strain-dependent modulation of collagen degradation that could influence overall alignment under uniaxial loading by selectively degrading fibers with certain orientations faster than others [49–51]. While all these effects could be present within the actual tendon, we found that strain-dependent cell alignment, deposition of collagen aligned with the cells, and strain-dependent changes in collagen synthesis rate were sufficient for capturing the major trends in the data as outlined above.

### Conclusions

In this study, we used multiscale modeling to integrate information from the literature on fibroblast responses to stretch (alignment and collagen synthesis), scar formation following injury (collagen deposition and other features of the agent-based model), and musculoskeletal mechanics (rat hindlimb model implemented in OpenSim) to interpret apparently conflicting data from a range of experimental studies. We found that our computational model could reproduce several key features of observed tendon healing across a wide array of reported experiments in rats–including the paradoxical finding that repairing transected tendon reverses the effect of loading on alignment–without fitting model parameters to any data from those experiments. Rather, the key to the predictions in our multiscale model was simulating the specific loading and surgical protocols to predict tissue-level strains, which then guided cellular behaviors according to response curves based on *in vitro* experiments. These results suggest that the apparently conflicting data in the studies we reviewed may in fact reflect consistent biologic responses to local strains in the healing tendon, providing a new conceptual framework for interpreting existing data and devising potential therapies for Achilles tendon rupture.

## Methods

### Literature collection

We searched PubMed and Google Scholar for all papers that included the keywords “rat Achilles tendon rupture injury” in the title or abstract and used a scalpel to perform a full transection of both the Achilles tendon and the plantaris tendon, a tendon parallel to the Achilles that is proportionally larger in rats than in humans and can act as an “internal splint” [52]. From these, we next identified studies that specifically compared the effects of unloading from intramuscular injection of Botox, tail suspension, or cast immobilization against loading due to free cage activity or treadmill exercise. From these 30 identified papers, we then selected those that quantitatively measured the cross sectional area and/or the Young’s modulus of the healing scar, leaving us with 10 studies that met our search criteria [10–14,24–26,36,53]. We used cross sectional area (CSA) of the tendon as a surrogate measure for collagen production, based on previous studies showing that collagen concentration and tendon CSA rise in parallel [24,29,30], as well as the fact that tissue mass increases with tissue volume. Similarly, we used the Young’s modulus as a surrogate measure for collagen alignment based on the strong reported correlation between measured alignment and intrinsic material properties of the tissue [6,14], and the fact that Young’s modulus was commonly measured in the studies we found while collagen alignment was not.

### OpenSim simulations and strains

We adapted a previously published rat right hindlimb model to conduct our simulations (Fig 1A) [16] (https://simtk.org/projects/rat_hlimb_model). Briefly, the model used anatomically accurate representations of the bones (spine, hip, femur, tibia, and foot), joints, and musculo-tendon units (each represented by one line segment from its origin to insertion) of the rat right hindlimb. Musculo-tendon units were represented as linear elements consisting of a muscle segment in series with a passive tendon segment. The muscle segment consisted of its own passive element in parallel with an active contractile element that generated force depending on a Hill-type model. The mechanical properties of muscle fiber and tendon were defined using fiber force-length, fiber force-velocity, and tendon force-strain curves determined by Millard et al. [54]. Muscle segments could be prescribed activation levels varying from 0 (rest) to 1 (full activation). Since muscles not attached to the Achilles tendon were irrelevant for this simulation, we simplified the model by only including the lateral and medial gastrocnemius and soleus muscles, the three musculo-tendon units that comprise the Achilles tendon. To predict Achilles tendon strains for the various cases simulated here, we prescribed experimentally measured joint angle profiles, muscle activation curves, and tendon mechanical properties as inputs, and obtained strain vs. time curves from forward dynamic simulations using custom MATLAB routines employed by two of the authors in a previous publication [55].

#### Passive tendon properties

To estimate physiologic strains for a ruptured tendon, we had to prescribe tendon parameters that reflected the early stages of healing. First, we decreased the slope of the linear portion of the tendon stress-strain curve by a factor of 5x, mimicking the decrease in material properties from a healthy tendon (~175 MPa) to an initial callus (~30 MPa) [24]. Next, we mathematically estimated tendon slack length (TSL) values using a previously published numerical optimization algorithm [56] that has also been previously utilized by two of the authors [55] and by Charles et al. [57]. The TSL is a value that represents the length at which the tendon begins to develop passive elastic force but is difficult to directly measure experimentally. Since the TSL must be a constant value for each tendon, if we know the optimal fiber length, the musculo-tendon lengths, and the normalized fiber lengths across a physiologic range of joint angles, we can numerically converge upon one TSL estimate using this algorithm. We used the calculated TSL values for our suture repaired tendons, since we assumed that suturing the two tendon stumps back together would form a ~0mm gap distance. In unrepaired tendons, we simulated the presence of a gap distance between the stumps by adding 7mm to each calculated TSL value, effectively shifting its reference (undeformed) length by 7mm. We used a gap distance of 7mm based on 3 separate studies that excised a 3mm section of the Achilles tendon and measured the gap distance between the stumps following excision to be ~10mm (14,19,20).

#### Prescribing loading and unloading using forward dynamic simulations

**Fig 8:**
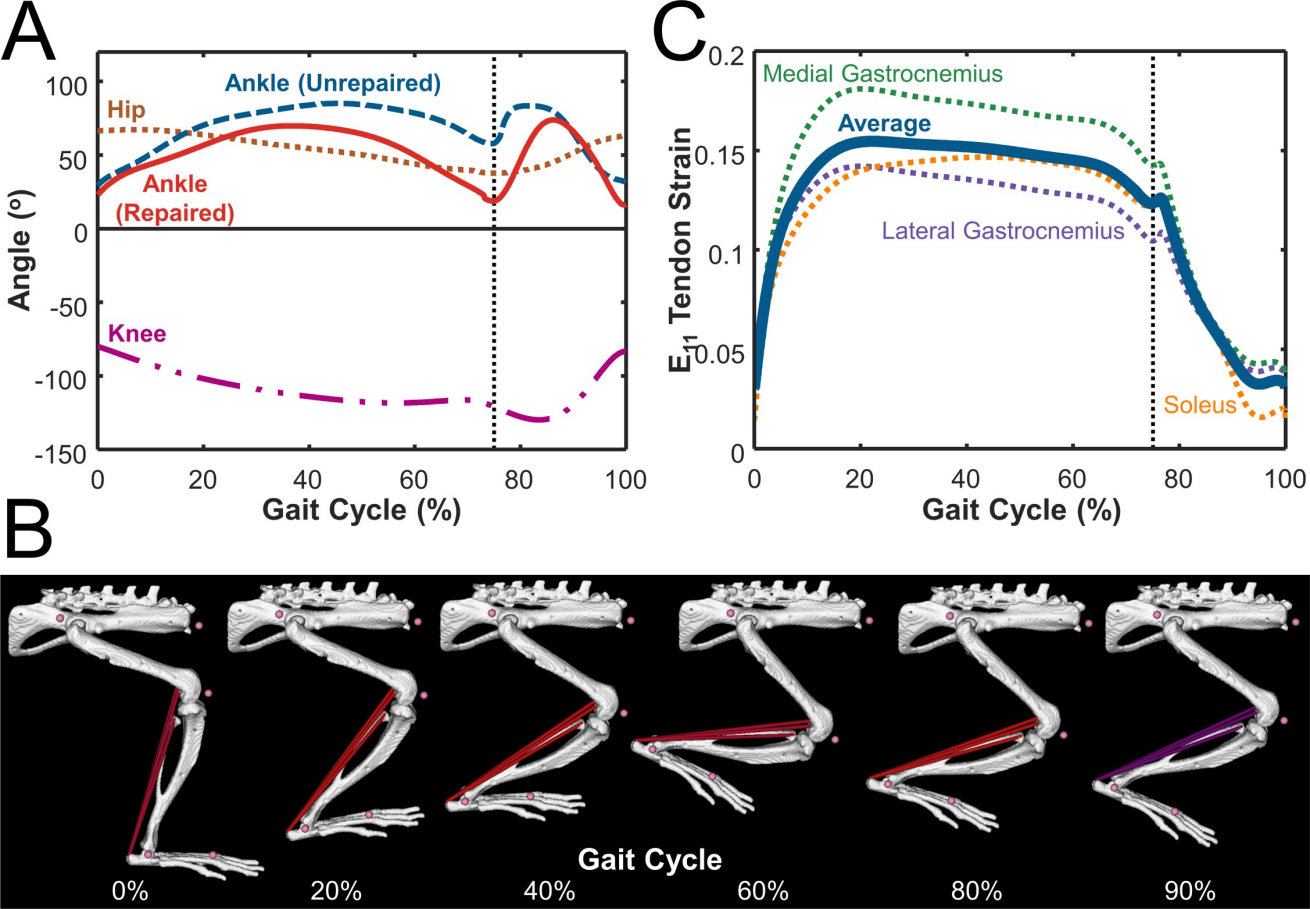
Rat hindlimb model implemented in OpenSim used joint angles, muscle activation, and passive tendon properties as inputs to determine tendon strains. **(A)** Hip (**brown**) and knee (**magenta**) angle data from healthy rat gait measured by Garnier et al. [41] and ankle angle data measured by Liang et al. [42] in both unrepaired (**blue**) and suture-repaired (**red**) tendons. Black dashed line separates stance phase (first 75%) from swing phase (last 25%). **(B)** Depiction of model motion during the prescribed gait cycle. The color of the muscle fiber depicts the activation during that part of the gait cycle, ranging from maximum activation (1, red) to minimum activation (0, blue). Purple fibers (shown during the swing phase at 90%) depict the transition from maximum to minimum activation. **(C)** Final tendon strain output was calculated by taking the average (**thick blue**) of tendon strains in the lateral (**purple**) and medial (**green**) gastrocnemius and soleus (**orange**) musculo-tendon units.

We categorized both free cage activity and treadmill exercise as loading conditions, since treadmill exercise conditions consisted of 23 hours of free cage activity + 1 hour of treadmill exercise prescribed at normal rat walking speeds [12,41]. We simulated loading by prescribing joint kinematics associated with the daily locomotion of a rat during a gait cycle. We used healthy knee and hip joint angles measured by Garnier et al. [41], as well as injured ankle joint angle data measured by Liang et al. [42], who measured ankle motion following Achilles injury in both unrepaired and suture-repaired cases (Fig 8A). We limited the degrees of freedom of each joint to the saggital plane, matching the plane of motion in the experimental flexion/extension data. All other available joints (e.g. ankle adduction/abduction, hip internal/external rotation, etc.) were held constant at the original rat hindlimb model angles determined for a healthy rat by Johnson 2008 [16]. We used stance and swing phases that comprised 75% and 25% of the total gait cycle [41], respectively. Based on studies conducted by Nicolopoulos‐Stournaras et al. [58], we prescribed a maximum activation value of 1 to all three musculo-tendon units during the stance phases and minimum activation of 0.05 during the swing phases. Fig 8B shows the model at multiple phases of the gait cycle. For unloading conditions, we assumed that intramuscular botox injection, tail suspension, or cast immobilization of the tendon would leave the rats unable to move their knee and ankle on the injured side and unable to activate any of the muscles connected to the Achilles. With these assumptions, we fixed the knee joint at -50^o^ flexion and the ankle at -30^o^ plantar flexion [12,13], with all other joints held at original model angles. We prescribed a constant minimal muscle activation value of 0.05 to avoid numerical singularity in the muscle model when activation approaches 0 [54].

#### Outputs

We used the model-predicted changes in tendon lengths over time to calculate tendon strains as (tendon length–tendon slack length) / (tendon slack length). We calculated the strains over time for each of the tendon segments and averaged them to determine the final tendon strain (Fig 8C).

### Thermodynamic computational model of cell alignment

We used a computational model of stress fiber remodeling published previously by our group to estimate cell alignment behavior [18] (Fig 1B). The model represents the thermodynamics of stress fiber (SF) assembly and disassembly, capturing features such as the ability of tension to promote assembly by altering the free energy of bound actin subunits. Two specific features of the model are important for the predictions shown in this manuscript. First, on the time scale of individual loading cycles, large negative strain rates reduce stress fiber tension through the force-velocity behavior of myosin, promoting SF disassembly in the direction of stretch and net SF (and cell) orientation perpendicular to that stretch. On a longer time scale, the model assumes that cells can remodel the extracellular matrix and/or their attachments to the ECM to attain an equilibrium strain state that minimizes the sum of the energies associated with elastic stretch and the chemical potential of the bound and unbound actin subunits. This aspect of the model drives the response to mean boundary conditions, upon which cyclic responses are then superimposed. Here, we used the average and spread of predicted SF distributions as surrogate measures of cell alignment, the same approach we used in the original model validation against *in vitro* data. For a more complete description of the model and details on its validation, please see Chen et al. 2018 [18].

#### Integration of cell alignment model into the multiscale model

We used a uniaxial stretch boundary condition in all cellular simulations, prescribing stretch (or constraint, in the unloaded case) in one direction (x_1_) while the transverse direction remained free (x_2_). We estimated the strains experienced by individual cells as 0.28 times the tissue-level tendon strains computed in OpenSim. This empirically determined correction factor accounts for two effects we expect to reduce actual cell strains below the tissue strains we calculated here. First, in many tissues the surrounding matrix partially shields cells from strain; for example, Screen et al. [59] stretched rat tail tendons and observed cell nuclear strain to be about half of the imposed tendon strains. Second, due to lack of data on the actual magnitude of muscle activation during the rat gait cycle, we prescribed maximum (= 1) activation to the muscle during stance phases in the rat hindlimb model [58]. In reality, it seems improbable that these muscles are maximally activating during the normal gait cycle. We then used the maximum and minimum estimated cellular E_11_ and frequency of stretch as inputs to calculate the alignment of stress fibers within the cell. We devised a hypothetical loading profile to simulate a rat walking around its cage freely, alternating 1-hour periods of 1Hz walking with 1-hour rest periods for 12 hours of waking, followed by 12 hours of no activity to simulate sleep, consistent with the 12h/12h light/dark cycles employed by almost all rat studies used for comparisons. In a typical rat gait cycle, injured rats walk with a gait cycle time of around 700ms [42], which corresponds to a stretch frequency between 1Hz and 2Hz; we chose the lower boundary of 1Hz. All other parameters in the cell alignment model remained unchanged from Chen et al. [18], and the model generated a predicted stress fiber orientation distribution for each strain state simulated. In order to quantitatively compare these stress fiber distributions, we used an order parameter [18,60,61]:

S=<cos2θ>=∫h(θ)cos(2θ)dθ, (1)

where *h*(*θ*) represents the probability distribution histogram of SFs in each angular bin. S ranges from S = 1, all cells or stress fibers aligned completely parallel to the stretch (x_1_) direction, to S = -1, all cells or fibers aligned completely perpendicular to stretch, with S = 0 representing completely random alignment.

### Agent based model of Achilles tendon wound healing

**Fig 9:**
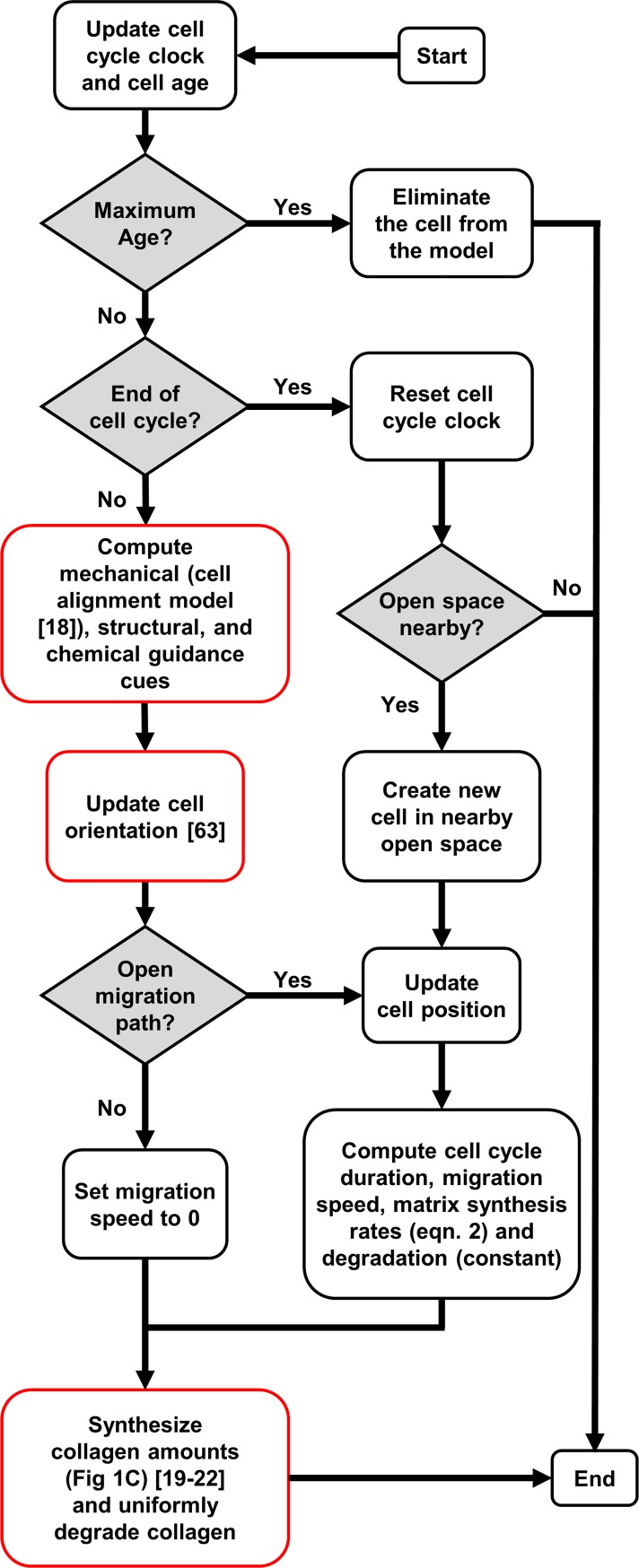
Flowchart adapted from Rouillard and Holmes [23] depicting the various decisions cells made in the agent-based model. Red outlines indicate modifications introduced in the current study.

**Fig 10:**
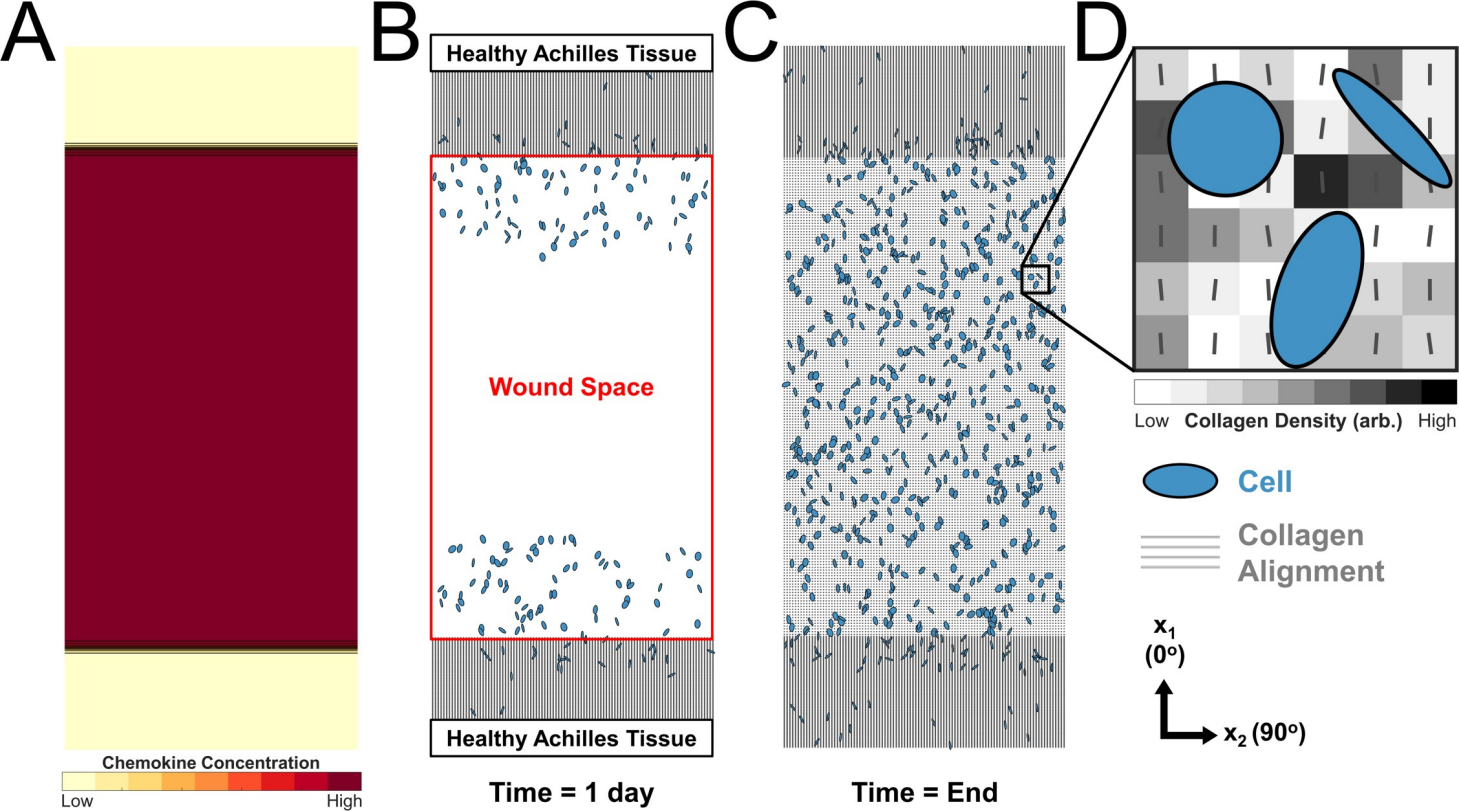
Schematic of the agent-based model of wound healing in the injured rat Achilles tendon. **(A)** Chemokine difference between the wound space and surrounding tissue drives cell migration into the wound. **(B)** Healthy Achilles tendon adjacent to the top and bottom of the wound space was comprised of fibroblasts (**blue ovals**) and highly aligned collagen (**grey**). The wound space was initially cell-free. **(C)** Cells migrated, proliferated, and synthesized and deposited collagen to create scar tissue within the wound area. **(D)** Magnification of the **boxed** area in **(C)** shows that the cells have different alignments and shapes determined by the cell alignment model and interact with 10μm x 10μm collagen patches (**grey boxes**). Each patch stored information on local collagen density (**grayscale tone**) and collagen alignment (**lines**). Cell sizes have been increased for visibility in this schematic.

We adapted an agent based model (ABM) originally published by Rouillard and Holmes [23,62] for infarct healing to integrate cellular-level responses and predict the evolving tendon scar structure (Fig 1D). Table 1 lists all model parameters altered for these simulations, while Fig 9 shows a flowchart of a cell’s decision tree within the model. Rouillard and Holmes modeled fibroblasts as circular discs free to move in a square, two-dimensional space divided into 10-micron-square patches. Each patch contained information about the local collagen alignment and density. Fibroblasts could migrate, proliferate, undergo apoptosis, and remodel collagen. Fibroblast orientation guided fibroblast migration direction and deposition of collagen, and existing collagen fibers were degraded at a rate proportional to their local concentration. A local chemokine concentration gradient with a high concentration of chemokines within the wound area and a low chemokine concentration in the healthy tissue drove cell migration into the wound (Fig 10A). We made several modifications to this model to adapt it for Achilles tendon healing. We simulated healing of a rectangular wound area after complete transection or rupture (Fig 10B). The initial wound size, matrix structure, and collagen content within the wound depended on the healing condition (Table 1). An unrepaired transected tendon contained a low amount (0.1%) of randomly aligned collagen fibers, mimicking the randomly aligned provisional matrix in the wound area, while a suture-repaired tendon contained some aligned collagen (0.9%, alignment order parameter of 0.4), mimicking aligned collagen fibers from the healthy tendon stumps. Fibroblasts migrated into the wound space (Fig 10B, 10C and 10D) from the two opposing sides adjacent to healthy tissue. Rouillard determined cell alignment from a series of phenomenologic equations that represented the alignment response to stretch, contact guidance from surrounding collagen, and chemokine gradients, as well as their integration. Here, we replaced the original phenomenologic relationship governing stretch-induced alignment with the cell alignment predicted from the stress fiber model, and employed a more recent equation for integrating across the alignment cues published by Richardson et al. [63].

**Table 1:** Parameters used in ABM.

Initial Wound	Value	Units
Width	1	mm
Length	0.5 (Repaired), 7 (Unrepaired)	mm
Collagen Fiber Area Fraction	0.001 (Unrepaired), 0.009 (Repaired)	
Collagen Fiber Alignment	0 (Unrepaired), 0.4 (Repaired)	see Eq 1
		
**Collagen**	**Value**	**Units**
Collagen Synthesis	See Fig 1C	collagen amount (arb.) per cell
Collagen Degradation	0.0025	h^-1^
Collagen Element Size	10x10	μm^2^
		
**Cell**	**Value**	**Units**
Cell Radius	5	μm
Cell Initial Density	500	cells/mm^2^
Time to apoptosis	240	hours
Time to mitosis	12 (quiescent), 240 (activated)	hours
Cell Speed	1 (quiescent), 50 (activated)	μm h^-1^

Based on previous studies showing that cells upregulate collagen production after exposure to both static [64] and cyclic stretch [32–35], cells synthesized collagen according to their mean strains, with higher strains corresponding to higher collagen synthesis rates. The mean strain was calculated by taking an average over one gait cycle period (1 second). In unloaded cases, this mean strain matched the static strain values (Fig 5). We determined the collagen synthesis amounts according to a sigmoidal curve fitted to data from four independent experiments in the literature (Fig 1C) [32–35]:

$$CollagenSynthesis(\varepsilon_{m})=\frac{1.3}{1+\exp\left(-150(\frac{\varepsilon_{m}}{2}-0.02)\right)}+0.6,$$ (2)

where *ε_m_* is the mean strain felt by the cell. Cells deposited collagen aligned to their major axis of alignment. While the exact mechanisms by which fibroblasts deposit and orient collagen *in vivo* are still being debated, the general idea that collagen ends up locally aligned with the fibroblasts that deposit it remains strongly supported in the literature [43–45]. At each time point for which collagen content and orientation are reported, the local collagen content and orientation histograms from each collagen patch were averaged to determine a single area fraction, mean angle, and order parameter for the entire scar region.
